# Depression and increased risk of non-alcoholic fatty liver disease in individuals with obesity

**DOI:** 10.1017/S204579602000116X

**Published:** 2021-03-12

**Authors:** In Young Cho, Yoosoo Chang, Eunju Sung, Jae-Heon Kang, Sarah H. Wild, Christopher D. Byrne, Hocheol Shin, Seungho Ryu

**Affiliations:** 1 Department of Family Medicine, Kangbuk Samsung Hospital, Sungkyunkwan University School of Medicine, Seoul, Republic of Korea; 2 Center for Cohort Studies, Total Healthcare Center, Kangbuk Samsung Hospital, Sungkyunkwan University School of Medicine, Seoul, Republic of Korea; 3 Department of Occupational and Environmental Medicine, Kangbuk Samsung Hospital, Sungkyunkwan University School of Medicine, Seoul, Republic of Korea; 4 Department of Clinical Research Design & Evaluation, SAIHST, Sungkyunkwan University, Seoul, Republic of Korea; 5 Usher Institute University of Edinburgh, Edinburgh, UK; 6 Nutrition and Metabolism, Faculty of Medicine, University of Southampton, Southampton, UK; 7 National Institute for Health Research Southampton Biomedical Research Centre, University Hospital Southampton, Southampton, UK

**Keywords:** Depression, hepatic fibrosis, hepatic steatosis, non-alcoholic fatty liver disease, obesity

## Abstract

**Abstract**

**Aims:**

The longitudinal relationship between depression and the risk of non-alcoholic fatty liver disease is uncertain. We examined: (a) the association between depressive symptoms and incident hepatic steatosis (HS), both with and without liver fibrosis; and (b) the influence of obesity on this association.

**Methods:**

A cohort of 142 005 Korean adults with neither HS nor excessive alcohol consumption at baseline were followed for up to 8.9 years. The validated Center for Epidemiologic Studies-Depression score (CES-D) was assessed at baseline, and subjects were categorised as non-depressed (a CES-D < 8, reference) or depression (CES-D ⩾ 16). HS was diagnosed by ultrasonography. Liver fibrosis was assessed by the fibrosis-4 index (FIB-4). Parametric proportional hazards models were used to estimate the adjusted hazard ratios (aHRs) and 95% confidence intervals (CIs).

**Results:**

During a median follow-up of 4.0 years, 27 810 people with incident HS and 134 with incident HS plus high FIB-4 were identified. Compared with the non-depressed category, the aHR (95% CIs) for incident HS was 1.24 (1.15–1.34) for CES-D ⩾ 16 among obese individuals, and 1.00 (0.95–1.05) for CES-D ⩾ 16 among non-obese individuals (*p* for interaction with obesity <0.001). The aHR (95% CIs) for developing HS plus high FIB-4 was 3.41 (1.33–8.74) for CES-D ⩾ 16 among obese individuals, and 1.22 (0.60–2.47) for CES-D ⩾ 16 among non-obese individuals (*p* for interaction = 0.201).

**Conclusions:**

Depression was associated with an increased risk of incident HS and HS plus high probability of advanced fibrosis, especially among obese individuals.

## Introduction

Non-alcoholic fatty liver disease (NAFLD) is a global health problem with a prevalence of approximately 25% worldwide (Maurice and Manousou, 2018). The prevalence of NAFLD-related advanced hepatic fibrosis, which leads to increased mortality risk, is also steadily increasing (Kim *et al*., 2019a). NAFLD is predicted to become the most common indication for liver transplantation in the following decade (Maurice and Manousou, 2018). However, there is no licensed pharmacological treatment for NAFLD, and lifestyle modification with weight loss continues to be the mainstay of NAFLD management, underscoring the importance of identifying modifiable risk factors for NAFLD and its progression (Maurice and Manousou, 2018).

Depression is a leading cause of disability worldwide (World Health Organization, 2017), with a lifetime prevalence of 6.5–21.0%. Depression also imposes a great burden on public health worldwide (Kessler and Bromet, 2013). A growing body of evidence suggests that depression is associated with increased risk of mortality, as well as various chronic diseases including obesity, hypertension, diabetes, coronary artery disease and stroke. All of these comorbidities are also commonly accompanied by NAFLD (Luppino *et al*., 2010; Dong *et al*., 2012; Cuijpers *et al*., 2014; Lichtman *et al*., 2014). However, the specific relationship between depression and NAFLD remains uncertain. Previous studies addressing this subject have reported inconsistent results, varying from a positive association (Jung *et al*., 2019; Kim *et al*., 2019b) to no evidence of an association (Surdea-Blaga and Dumitrascu, 2011; Lee *et al*., 2013). However, many of these prior studies were limited by small sample sizes, ambiguous temporality due to cross-sectional designs and various definitions of NAFLD, depression and control groups. Previous studies on NAFLD have also shown that depression is associated with a higher steatosis grade and more severe histology (Youssef *et al*., 2013; Tomeno *et al*., 2015). In addition, obesity is closely associated with both NAFLD and depression (Buzzetti *et al*., 2016; Jantaratnotai *et al*., 2017), and may act as a mediator or effect modifier in the relationship between depression and NAFLD. Depression and obesity often co-exist (Jantaratnotai *et al*., 2017), and previous studies have suggested a synergistic effect of the co-existence of depression and obesity on adverse health outcomes such as coronary heart disease risk (Ladwig *et al*., 2006; Yakar and Ertekin, 2018).

Until now, no longitudinal cohort studies have investigated the effect of depression on NAFLD development and tested effect modification by obesity. Therefore, in obese and non-obese subjects without hepatic steatosis (HS) who had a low probability of liver fibrosis at baseline, we examined whether depression is associated with: (a) an increased risk of incident HS and (b) HS plus high probability of advanced fibrosis.

## Methods

### Study population

The Kangbuk Samsung Health Study is a cohort including Korean men and women who received comprehensive annual or biennial health examinations at the Seoul and Suwon Kangbuk Samsung Hospital Total Healthcare Centers (Chang *et al*., 2016; 2019). In South Korea, the Industrial Safety and Health Law mandates annual or biennial health screening exams for employees. The study population participated in health screening examinations from March 2011 to December 2017, and had at least one follow-up visit by 31 December 2019 (*N* = 286 969). More than 80% of participants were employees of companies or local governmental organisations, or their spouses, while the remaining participated voluntarily in the health check-up programmes.

**Fig. 1.**
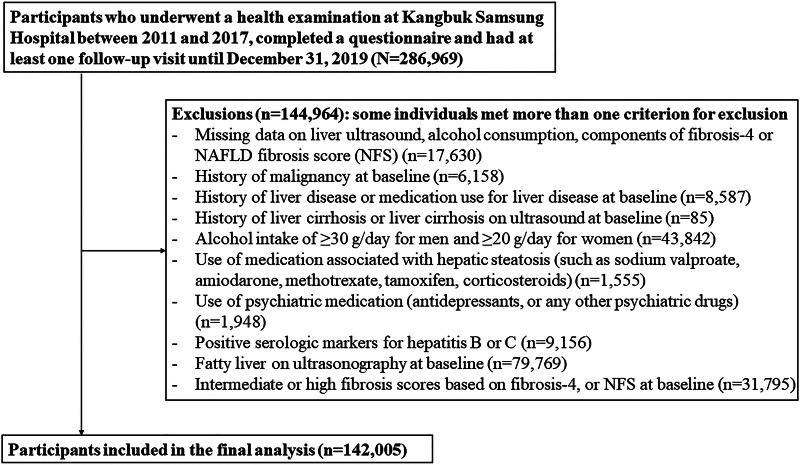
Flowchart of study participants.

A total of 144 964 subjects met one or more exclusion criteria at baseline (Fig. 1), and men who consumed alcohol ⩾30 g/day and women who consumed ⩾20 g/day were also excluded. The final sample included 142 005 participants. This study was approved by the Institutional Review Board of Kangbuk Samsung Hospital (IRB 2020-04-007) and conformed to the 1964 Declaration of Helsinki and its later amendments. The requirement for informed consent was waived due to the use of a preexisting de-identified dataset that was routinely collected during the health screening process.

### Data collection

Data regarding demographic and behavioural factors, medical history and current medication were collected using standardised self-administered questionnaires. Participants were categorised as never, former or current smokers. Current alcohol consumption was assessed by the frequency of alcohol consumption per week and the amount of alcohol intake per drinking day. The frequency and amount consumed per drinking day were then used to calculate average alcohol consumption per day. The level of physical activity was determined through the validated Korean version of the International Physical Activity Questionnaire (IPAQ) Short Form (Craig *et al*., 2003; Chun, 2012). Health-enhancing physical activity (HEPA) was defined when one of the two following criteria were met: (1) vigorous activity on ⩾3 days/week with ⩾1500 of accumulated metabolic equivalent (MET)-minutes/week (1 MET is equivalent to energy expenditure at rest); or (2) 7 days of walking, moderate intensity or vigorous intensity activities combined resulting in ⩾3000 MET min/week (Craig *et al*., 2003). Usual dietary consumption during the past year was assessed using a 106-item Korean version of self-administered food frequency questionnaire (Ahn *et al*., 2007). The 20-item Korean version of the Center for Epidemiological Studies-Depression (CES-D) was used to evaluate depressive symptoms, for which the internal consistency has been reported to range from 0.84 to 0.91 (Cho and Kim, 1998). The presence of clinically significant depressive symptoms was defined at baseline as a CES-D score ⩾16, which has been established and validated as identifying depression in previous studies (Beekman *et al*., 1997; Cho *et al*., 1998). The non-depressed group (identified as CES-D < 8) was used as the reference because the negative impact of less severe depressive symptoms (described as sub-threshold depression) on various health outcomes has been reported compared to a non-depressed group (Hybels *et al*., 2001; Vahia *et al*., 2010; Cuijpers *et al*., 2013). Thus, for this study, the CES-D scores were categorised as non-depressed (CES-D < 8), sub-threshold depression (CES-D: 8–15) and depression (CES-D ⩾ 16) (Hybels *et al*., 2001; Vahia *et al*., 2010; Cuijpers *et al*., 2013).

Patient seated blood pressure (BP), height and weight measurements were obtained by trained nurses. Obesity was defined as body mass index (BMI) ⩾ 25 kg/m^2^, the proposed cutoff for obesity diagnosis in Asians (World Health Organization and Regional Office for the Western Pacific, 2000). Hypertension was identified through systolic BP ⩾140 mmHg, diastolic BP ⩾90 mmHg, or the use of any antihypertensive medications.

### Laboratory analyses

Blood specimens were obtained after fasting for >10 h. The blood measurements included the following: total cholesterol, low-density lipoprotein cholesterol (LDL-C), high-density lipoprotein cholesterol (HDL-C), triglycerides, glucose, insulin, albumin, aspartate aminotransferase (AST), alanine aminotransferase (ALT), gamma-glutamyltransferase and platelets (Chang *et al*., 2016). Insulin resistance was evaluated using the homoeostatic model assessment-insulin resistance (HOMA-IR) equation as follows: fasting blood insulin (μU/ml) × fasting blood glucose (FBG) (mmol/l)/22.5. Diabetes mellitus was defined as FBG ⩾126 mg/dl, haemoglobin A1c (HbA1c) ⩾6.5% or current use of anti-diabetic medication.

The diagnosis of HS was based on abdominal ultrasounds performed by experienced radiologists blinded to the aim of this study. Diagnosis was based on standard criteria: diffuse increase of fine echoes in the liver parenchyma compared with the kidney or spleen parenchyma, deep beam attenuation and bright vessel walls (Mathiesen *et al*., 2002). Inter- and intra-observer reliability values for diagnoses of HS were substantial (kappa statistic of 0.74) and excellent (kappa statistic of 0.94), respectively (Kim *et al*., 2013, 2019c; Chang *et al*., 2018).

To evaluate the risk of more severe HS, we used two non-invasive indices of liver fibrosis: the fibrosis-4 (FIB-4) score, and the NAFLD fibrosis score (NFS) (Angulo *et al*., 2007; Shah *et al*., 2009). The FIB-4 index was calculated as FIB-4 = (age (years) × AST (U/l))/(platelet count ( × 10^9^/l) × ALT (U/l)^1/2^) (Shah *et al*., 2009). The NFS was calculated as NFS = −1.675 + 0.037 × age (years) + 0.094 × BMI (kg/m^2^) + 1.13 × impaired fasting glucose or diabetes (yes = 1, no = 0) + 0.99 × AST/ALT ratio − 0.013 × platelet ( × 10^9^/l) − 0.66 × albumin (g/dl) (Angulo *et al*., 2007). FIB-4 of ⩾2.67 or NFS of >0.676 cutoff values were used to identify high probability of advanced liver fibrosis, and FIB-4 of 1.30 to 2.66 or NFS of 0.676 to −1.455 were used to identify intermediate probability of advanced fibrosis (Angulo *et al*., 2007; Shah *et al*., 2009).

Clinical and biochemical components of FIB-4, NFS and HS based on abdominal ultrasound were collected as part of basic health check-up programmes and thus FIB-4, NFS and HS could be annually or biennially evaluated at each baseline and follow-up visit for this study.

### Statistical analysis

The baseline characteristics of the study participants were described according to the CES-D category. Since there was a difference in age and sex between those with depression and those without, the baseline characteristics were presented as age- and sex-adjusted means or proportions and 95% confidence intervals (CIs).

The primary endpoints were: (a) development of incident HS and (b) development of incident HS plus high probability of advanced fibrosis based on FIB-4 scores. Incident HS and incident HS plus high fibrosis score were treated as separate endpoints in each model. For analysis of the association between depression and incident HS, if HS occurred during follow-up, subsequent observations were not incorporated in further analysis. For analysis of the association between depression and incident HS plus high probability of advanced fibrosis, if an individual's ultrasonographic finding indicated HS and non-invasive fibrosis markers showed high probability of advanced fibrosis during follow-up, the case was assumed to have developed HS plus high probability of advanced fibrosis. The event detection date was defined as the earliest date of identification of HS or HS plus high fibrosis score. The person-years were computed as the sum of the follow-up duration from baseline to the event detection date (HS or HS plus high fibrosis score, separately) or until the final examination (before 31 December 2019), whichever occurred first. The incidence rates were computed as number of incident cases divided by the person-years of follow-up. Either primary endpoint would have occurred at an unknown time point between the event detection date and the previous screening visit. Therefore, we used a parametric proportional hazards model for interval censoring, and the baseline hazard function was parameterised with restricted cubic splines in log time using four degrees of freedom.

The risk of incident HS and HS plus high fibrosis score were separately evaluated according to the CES-D category. The hazard ratio (HR) and 95% CI were calculated using the parametric proportional hazards model. The models were initially adjusted for age and sex, and were then further adjusted for the following potential confounders: study centre (Seoul, Suwon), year of screening exam, smoking (never, past, current or unknown), alcohol intake (none, <10 g/day, ⩾10 g/day or unknown), physical activity (inactive, minimally active, HEPA or unknown), BMI (continuous), education level (<community college graduate, ⩾community college graduate or unknown), total energy intake, diabetes, hypertension and history of cardiovascular disease (CVD) (model 1). We next sought to examine whether the relationship between depression and development of the primary endpoints was mediated by insulin resistance, inflammation and metabolic abnormalities. Therefore, model 2 was further adjusted for HOMA-IR, high-sensitivity C-reactive protein (hs-CRP) and other metabolic factors including systolic BP, glucose, total cholesterol, HDL-C and triglycerides. We assessed the proportional hazards assumption by examining graphs of estimated log(−log(survival)) *v.* log of survival time graph: no violation of the assumption was found.

We then evaluated whether or not the associations between depressive symptoms and the risk of HS or HS plus high fibrosis score differed by the presence of obesity (as defined by BMI ⩾ 25 kg/m^2^) (World Health Organization and Regional Office for the Western Pacific, 2000), because the effect of depression on various clinical outcomes appears to be increased by the presence of obesity (Ladwig *et al*., 2006; Yakar and Ertekin, 2018). The interactions between the CES-D categories and obesity on the risk of HS, and the high probability of advanced liver fibrosis were tested using likelihood ratio tests. These tests compare models with and without multiplicative interaction terms. Sensitivity analyses were performed using NFS as another validated non-invasive liver fibrosis score. We also performed time-dependent analyses with CES-D score category, BMI, smoking status, alcohol consumption, physical activity, total energy intake, diabetes, hypertension and history of CVD as time-varying covariates. ‘*p* for linear trend’ was tested by including the CES-D categories as a continuous variable on each model.

The statistical analyses were performed using STATA version 16.0 (StataCorp LP, College Station, TX, USA). All reported *p* values were two-tailed. Differences with a *p* value <0.05 were considered statistically significant.

## Results

Table 1 shows the baseline characteristics of 142 005 participants by CES-D categories. The proportions of people with sub-threshold depression (CES-D; 8–15) and depression (CES-D ⩾ 16) were 25.6 and 11.9%, respectively (Table 1). Participants with depression were more likely to be younger and female than the non-depressed. After adjusting for age and sex, depression was positively associated with current smoking, alcohol intake, diabetes, hypertension, history of CVD, total calorie intake and slightly elevated glucose and triglyceride levels. In contrast, depression was inversely associated with the educational level.

**Table 1.** Baseline characteristics^a^ stratified by CES-D score category (*n* = 142 005)

Characteristics	CES-D score category			*p* value for trend
	<8	8–15	⩾16	
Number	88 655	36 389	16 961	
Age (years)	35.9 (35.9–35.9)	36.1 (36.0–36.1)	34.8 (34.7–34.9)	<0.001
Male (%)	44.6 (44.3–44.9)	37.9 (37.4–38.4)	23.6 (22.9–24.2)	<0.001
Current smoker (%)	12.6 (12.4–12.8)	15.2 (14.8–15.6)	17.9 (17.2–18.5)	<0.001
Alcohol intake (%)^b^	24.6 (24.4–24.9)	26.4 (26.0–26.8)	28.4 (27.7–29.0)	<0.001
HEPA (%)	14.6 (14.4–14.9)	14.2 (13.9–14.6)	14.3 (13.8–14.9)	0.100
High education level (%)^c^	88.9 (88.7–89.1)	82.4 (82.0–82.7)	81.2 (80.6–81.8)	<0.001
Hypertension (%)	4.2 (4.0–4.3)	4.2 (4.0–4.4)	4.5 (4.2–4.9)	0.085
Diabetes (%)	0.6 (0.5–0.6)	0.6 (0.5–0.7)	0.8 (0.6–0.9)	0.026
History of CVD (%)	0.4 (0.4–0.5)	0.6 (0.5–0.6)	0.8 (0.6–0.9)	<0.001
Medication for dyslipidaemia (%)	0.8 (0.7–0.8)	0.8 (0.7–0.8)	0.7 (0.6–0.8)	0.402
History of depression (%)	0.3 (0.3–0.4)	0.5 (0.4–0.5)	0.9 (0.8–1.1)	<0.001
Obesity (%)^d^	12.1 (11.9–12.3)	12.1 (11.7–12.4)	12.7 (12.2–13.3)	0.087
Body mass index (kg/m^2^)	21.9 (21.9–21.9)	21.9 (21.8–21.9)	21.9 (21.8–21.9)	0.819
Systolic BP (mmHg)	105.0 (104.9–105.1)	104.9 (104.8–105.0)	104.6 (104.4–104.7)	<0.001
Diastolic BP (mmHg)	66.9 (66.9–67.0)	67.0 (66.9–67.1)	66.9 (66.7–67.0)	0.979
Glucose (mg/dl)	91.2 (91.1–91.2)	91.3 (91.2–91.4)	91.3 (91.1–91.4)	0.026
Total cholesterol (mg/dl)	187.1 (186.8–187.3)	186.5 (186.2–186.8)	187.3 (186.9–187.8)	0.741
LDL-C (mg/dl)	113.4 (113.3–113.6)	112.8 (112.5–113.1)	113.3 (112.9–113.7)	0.031
HDL-C (mg/dl)	63.2 (63.1–63.3)	62.9 (62.7–63.0)	63.0 (62.8–63.2)	0.004
Triglycerides (mg/dl)	86.4 (86.1–86.7)	86.9 (86.5–87.4)	87.2 (86.5–87.9)	0.006
AST (U/l)	18.9 (18.9–18.9)	18.9 (18.8–18.9)	18.7 (18.6–18.8)	<0.001
ALT (U/l)	17.2 (17.2–17.3)	17.2 (17.1–17.3)	17.0 (16.9–17.2)	<0.001
GGT (U/l)	20.2 (20.1–20.4)	20.9 (20.7–21.1)	20.7 (20.4–21.0)	<0.001
Albumin (g/dl)	4.6 (4.6–4.6)	4.6 (4.6–4.6)	4.6 (4.6–4.6)	<0.001
Platelet (×10^9^/l)	250.3 (250.0–250.6)	250.7 (250.2–251.2)	252.8 (252.0–253.6)	<0.001
hs-CRP (mg/l)	0.8 (0.8–0.9)	0.9 (0.8–0.9)	0.9 (0.8–0.9)	0.914
HOMA-IR	1.18 (1.18–1.19)	1.19 (1.18–1.19)	1.20 (1.19–1.21)	0.853
Fib-4	0.70 (0.70–0.70)	0.70 (0.69–0.70)	0.69 (0.69–0.69)	<0.001
NFS	−3.24 (−3.25 to ~−3.24)	−3.23 (−3.24 to ~−3.22)	−3.25 (−3.27 to ~−3.24)	0.598
Total calorie intake (kcal/day)^e^	1114.3 (1108.7–1119.8)	1064.6 (1055.9–1073.3)	1158.4 (1145.6–1171.3)	<0.001

ALT, alanine aminotransferase; AST, aspartate aminotransferase; BP, blood pressure; CES-D, Center for Epidemiologic Studies-Depression Score; FIB-4, fibrosis-4; GGT, gamma-glutamyl transpeptidase; HDL-C, high-density lipoprotein-cholesterol; hs-CRP, high sensitivity C-reactive protein; HEPA, health enhancing physical activity; HOMA-IR, homoeostasis model assessment of insulin resistance; LDL-C, low-density lipoprotein cholesterol; NFS, non-alcoholic fatty liver disease fibrosis score.

a Age- and sex-adjusted mean or proportion with 95% CIs.

b ⩾10 g of ethanol per day.

c ⩾College graduate.

d BMI ⩾ 25 kg/m^2^.

e Among 105 083 participants with a plausible estimated energy intake level (within three standard deviations of the log-transformed mean energy intake).

During a median follow-up of 4.0 years (interquartile range, 2.2–6.0 years; maximum, 8.9 years; a total of 606 576.2 person-years), 27 810 participants developed HS (incidence rate, 45.8 per 1000 person-years) and 134 participants developed HS plus high FIB-4 (incidence rate, 20.0 per 100 000 person-years). Depression was weakly and positively associated with an increased risk of HS, but this association was more evident in obese individuals than it was in non-obese individuals (Table 2). After adjusting for age, sex, centre, year of screening exam, education level, BMI, smoking status, physical activity, total energy intake, diabetes, hypertension and history of CVD, the adjusted HR (95% CI) for incident HS comparing CES-D ⩾ 16 to CES-D < 8 was 1.24 (1.15–1.34), among participants with obesity, whereas the corresponding adjusted HR (95% CI) was 1.00 (0.95–1.05) among those without obesity (*p* for interaction by obesity < 0.001). After further adjustment for HOMA-IR, hs-CRP and other metabolic factors (including systolic BP, glucose, total cholesterol, HDL-cholesterol and triglycerides), these associations were similarly observed.

**Table 2.** Cumulative incidence rates and risk of incident HS according to CES-D score category in all, non-obese and obese individuals

CES-D score category	Person-years (PY)	Incident cases	Incidence (per 10^3^ PY)	Cumulative incidence (per 10^3^ person)		Age-sex aHR^a^ (95% CI)	Multivariable aHR^a^ (95% CI)	
				2-Years	5-Years		Model 1	Model 2
Overall (*n* = 142 005)								
<8	375 354.4	17 819	47.5	70.1	212.9	1.00 (reference)	1.00 (reference)	1.00 (reference)
8–15	158 046.5	7232	45.8	65.3	203.3	1.02 (1.00–1.05)	1.03 (1.00–1.06)	1.03 (1.00–1.06)
⩾16	73 175.3	2759	37.7	50.8	166.9	1.07 (1.03–1.12)	1.06 (1.01–1.10)	1.07 (1.03–1.11)
*p* for trend						<0.001	0.002	<0.001
Non-obese (*n* = 124 794)								
<8	335 387.9	12 728	38.0	52.8	171.6	1.00 (reference)	1.00 (reference)	1.00 (reference)
8–15	142 561.0	5243	36.8	49.3	162.9	1.02 (0.99–1.05)	1.02 (0.99–1.06)	1.04 (1.00–1.07)
⩾16	66 881.6	1985	29.7	39.1	129.7	1.01 (0.96–1.06)	1.00 (0.95–1.05)	1.02 (0.98–1.07)
*p* for trend						0.401	0.502	0.072
Obese (*n* = 17 211)								
<8	39 966.4	5091	127.4	188.4	495.0	1.00 (reference)	1.00 (reference)	1.00 (reference)
8–15	15 485.6	1989	128.4	184.3	502.7	1.05 (0.99–1.10)	1.05 (1.00–1.11)	1.03 (0.98–1.09)
⩾16	6293.7	774	123.0	153.3	486.8	1.25 (1.16–1.35)	1.24 (1.15–1.34)	1.25 (1.16–1.35)
*p* for trend						<0.001	<0.001	<0.001

BMI, body mass index; CES-D, Center for Epidemiologic Studies-Depression Score; CI, confidence interval; aHR, adjusted hazard ratio; HDL-C, high density lipoprotein cholesterol; HOMA-IR, homoeostasis model assessment of insulin resistance; hs-CRP, high sensitivity C-reactive protein.

a Estimated from parametric proportional hazard models. Multivariable model 1 was adjusted for age, sex, centre, year of screening exam, education level, BMI, smoking status, physical activity, total energy intake, diabetes, hypertension and CVD; model 2: model 1 plus adjustment for systolic blood pressure, glucose, total cholesterol, triglyceride, HDL-C, HOMA-IR and hs-CRP.

*Note*: *p* < 0.001 for the overall interaction between obesity and CES-D score category for incident HS (model 1).

Table 3 shows the risk of incident HS plus high FIB-4 according to depression and obesity. The association between depression and the risk of HS plus high FIB-4 tended to be stronger among obese individuals than non-obese individuals, although the difference was not statistically significant (*p* for interaction = 0.201). The multivariable adjusted HR (95% CI) for the development of HS plus high FIB-4 comparing CES-D ⩾ 16 to CES-D of <8 were 3.41 (1.33–8.74), among participants with obesity, whereas the corresponding HRs (95% CI) among non-obese individuals were 1.22 (0.60–2.47). Further adjustment for HOMA-IR, hs-CRP and other metabolic factors did not change qualitatively. These patterns were similarly observed in the sensitivity analyses using a high NFS of >0.676 instead of high FIB-4 (online Supplementary Table 1). In a sensitivity analysis for HS plus high FIB-4 by using FIB-4 cutoff score of 3.25, as recommended by the American Association for the Study of Liver Diseases guidelines (Chalasani *et al*., 2018), the associations between depression and risk of HS plus high FIB-4 were consistently observed in the obese group (online Supplementary Table 3). In time-dependent analyses where CES-D scores and other confounders were updated as time-varying covariates, the risk for HS remained highest in obese subjects with depressive symptoms while the association between CES-D categories and risk of HS plus high fibrosis score was no longer significant (online Supplementary Table 2).

**Table 3.** Cumulative incidence rates and risk of incident HS plus high probability of advanced fibrosis based on FIB-4 according to CES-D score category in all, non-obese and obese individuals

CES-D score category	Person-years (PY)	Incident cases	Incidence (per 10^3^ PY)	Cumulative incidence (per 10^3^ person)		Age-sex aHR^a^ (95% CI)	Multivariable aHR^a^ (95% CI)	
				2-Years	5-Years		Model 1	Model 2
Overall (*n* = 142 005)								
<8	415 497.9	77	0.2	0.1	0.5	1.00 (reference)	1.00 (reference)	1.00 (reference)
8–15	174 664.8	42	0.2	0.1	0.5	1.35 (0.93–1.97)	1.38 (0.94–2.02)	1.39 (0.95–2.03)
⩾16	78 787.3	15	0.2	0.1	0.3	1.72 (0.98–3.00)	1.70 (0.97–2.98)	1.68 (0.96–2.95)
*p* for trend						0.388	0.026	0.026
Non-obese (*n* = 124 794)								
<8	363 621.6	61	0.2	0.1	0.4	1.00 (reference)	1.00 (reference)	1.00 (reference)
8–15	154 299.8	30	0.2	0.1	0.4	1.21 (0.78–1.87)	1.24 (0.80–1.93)	1.26 (0.81–1.95)
⩾16	70 856.4	9	0.1	0.1	0.2	1.26 (0.62–2.55)	1.22 (0.60–2.47)	1.22 (0.60–2.47)
*p* for trend						0.349	0.357	0.340
Obese (*n* = 17 211)								
<8	51 876.3	16	0.3	0.1	0.7	1.00 (reference)	1.00 (reference)	1.00 (reference)
8–15	20 365.0	12	0.6	0.2	0.9	1.91 (0.90–4.03)	1.92 (0.91–4.08)	1.93 (0.91–4.10)
⩾16	7930.9	6	0.8	0.0	0.8	3.60 (1.41–9.20)	3.41 (1.33–8.74)	3.55 (1.38–9.13)
*p* for trend						0.005	0.007	0.005

BMI, body mass index; CES-D, Center for Epidemiologic Studies-Depression Score; CI, confidence interval; FIB-4, fibrosis 4 index; aHR, adjusted hazard ratio; HDL-C, high density lipoprotein cholesterol; HOMA-IR, homoeostasis model assessment of insulin resistance; hs-CRP, high sensitivity C-reactive protein.

a Estimated from parametric proportional hazard models. Multivariable model 1 was adjusted for age, sex, centre, year of screening exam, education level, BMI, smoking status, physical activity, total energy intake, diabetes, hypertension and CVD; model 2: model 1 plus adjustment for systolic blood pressure, glucose, total cholesterol, triglyceride, HDL-C, HOMA-IR and hs-CRP.

*Note*: *p* = 0.201 for the overall interaction between obesity and CES-D score category for incident HS plus high FIB-4 (model 1).

Although we excluded men and women who consumed alcohol ⩾30 and ⩾20 g/day, respectively at baseline, we also conducted a sensitivity analysis for HS and HS plus high fibrosis score after excluding subjects who reported binge drinking (⩾6 drinks per occasion). We found that depression remained positively associated with HS and HS plus high fibrosis score, especially among obese subjects (online Supplementary Table 4).

## Discussion

Our novel results in a cohort of 142 852 Korean young and middle-aged individuals demonstrate a longitudinal relationship between depression at baseline and the development of NAFLD, including both HS (diagnosed by ultrasound) and HS plus high probability of advanced fibrosis based on the use of two non-invasive fibrosis indices (FIB-4 and NFS). Our results also demonstrate that the associations appear to be more pronounced in individuals with obesity than in those without obesity, which suggests that obesity adversely affects the association between depression and NAFLD. Our findings suggest that depression may contribute to the development of HS and fibrosis, with a potentially synergistic effect of combined depression and obesity.

Previous studies have examined the association between depression and NAFLD. Some studies found a positive association between depression and NAFLD (Jung *et al*., 2019; Kim *et al*., 2019b), while others showed no association (Surdea-Blaga and Dumitrascu, 2011; Lee *et al*., 2013). However, the previous studies that found no association between depression and NAFLD were limited in the following ways: small sample size (Surdea-Blaga and Dumitrascu, 2011); the use of a NAFLD definition that was based on elevated ALT (Lee *et al*., 2013); the comparison of NAFLD subjects with individuals affected by other chronic liver diseases such as chronic hepatitis C or alcohol-related liver disease, rather than to those without liver disease (Surdea-Blaga and Dumitrascu, 2011; Lee *et al*., 2013). In contrast, in a cross-sectional study using a nationally representative sample of the United States population, depression was associated with 1.6–2.2-fold increased prevalence of NAFLD, but not with NAFLD-related advanced fibrosis (Kim *et al*., 2019b). In that study, after adjusting for diabetes, obesity and insulin resistance, the association between depression and NAFLD was attenuated but remained statistically significant, indicating that the association between depression and NAFLD may be mediated, in part, by metabolic factors including obesity (Kim *et al*., 2019b). Our cohort study extends these findings by demonstrating a longitudinal relationship between depression and HS plus high probability of advanced fibrosis based on non-invasive fibrosis indices, FIB-4 and NFS. Furthermore, our study also demonstrates that the association with HS alone and HS plus high fibrosis score were more pronounced in individuals with obesity than the non-obese. These results suggest that depression may adversely affect NAFLD, especially in the presence of obesity.

Our findings of the association between depression and HS plus high fibrosis score are in line with previous findings. One short-term follow-up study of 258 patients with biopsy-proven NAFLD demonstrated an association between depression, severe histological steatosis and higher NAFLD activity scores at baseline (Tomeno *et al*., 2015). Another cross-sectional study of 567 patients with biopsy-proven NAFLD showed that depression was associated in a dose-dependent manner with more severe hepatocyte ballooning (Youssef *et al*., 2013). However, the temporal relationship between depression and NAFLD severity remained unclear based on these prior studies. Findings from patients that underwent liver biopsy at university hospitals might not be generalisable to the low-risk or average-risk general population. Our study reveals an association between depression and the development of more severe HS plus high probability of advanced fibrosis in a generally healthy population using non-invasive biomarkers. This relationship was particularly true in the presence of obesity. This finding supports the notion that individuals with depression, but without NAFLD at baseline, may be at increased risk of developing different degrees of NAFLD (including both new-onset HS and HS plus high probability of advanced fibrosis). However, when changes in CES-D, obesity and other confounders were updated as time-varying covariates, the association between depression and risk of HS remained statistically significant, while the association with HS plus high fibrosis score did not; possibly longer time is required for depression to affect the progression to HS plus high probability of advanced fibrosis.

Although the underlying mechanisms between depression, NAFLD and obesity remain unknown, a physiological stress response affecting stress hormones, chronic inflammation, oxidative stress, and insulin resistance may be involved. The pathogenesis of NAFLD is not completely understood, but is thought to involve multiple insults that act together (Buzzetti *et al*., 2016). A body of evidence suggests that depression is associated with chronic low-grade inflammation, and increased inflammatory cytokine levels including tumour necrosis factor, interleukin-1*β* and interleukin-6 (Moylan *et al*., 2013; Miller and Raison, 2016; Kohler *et al*., 2017). Excessive activity of the hypothalamic–pituitary–adrenal axis, including elevated cortisol and corticotrophin-releasing hormone, is implicated in the pathogenesis of depression (Varghese and Brown, 2001; Keller *et al*., 2017). Insulin resistance, a key pathogenic feature, and its phenotypes (including type 2 diabetes and metabolic syndrome) have been reported to be associated with depression (Kan *et al*., 2013; Moulton *et al*., 2015; Chan *et al*., 2019). Another possible mechanism of the relationship between depression and NAFLD involves increased monoamine oxidase-A (MAO-A) activity, which has been identified in depressed patients. MAO-A is thought to augment cellular oxidative stress (Youssef *et al*., 2013). All of these characteristics, accompanied by depression, might contribute to the development of NAFLD and its progression. The role of obesity in the relationship between depression and NAFLD may also be explained by previous studies. Alterations in glucocorticoids, adipokines, insulin resistance and increased inflammatory mediators, including interleukin-6 and tumour necrosis factor alpha, are involved in obesity. Depression and obesity may interact to create a vicious cycle that potentiates NAFLD development (Kahn and Flier, 2000; Miller *et al*., 2003; Luppino *et al*., 2010; Hryhorczuk *et al*., 2013; Ellulu *et al*., 2017).

Depression may also increase the risk of HS, and HS plus high probability of advanced fibrosis through inadequate physical activity and consumption of an unhealthy diet. Currently, there are no approved treatments for NAFLD. Instead, it is managed through lifestyle modification (Maurice and Manousou, 2018). However, patients with NAFLD and major depressive disorder generally respond poorly to standard care (Tomeno *et al*., 2015). Therefore, it may be reasonable to screen and provide treatment for depression in patients at high risk for HS and advanced fibrosis. In this study, we adjusted for a wide range of covariates, including lifestyle behaviours (such as low level of alcohol intake, physical activity, smoking and total energy intake). Even after these adjustments, depression appeared to be independently associated with HS and HS plus high probability of advanced fibrosis.

Our study has several limitations. First, although histological diagnosis is the gold standard for liver disease, HS and liver fibrosis were assessed through ultrasound and two validated non-invasive markers of liver fibrosis in our study. Throughout the long period of follow-up, intra- and inter-observer agreement tests between the radiologists were not performed on a regular basis in our study. Different radiologists were involved over time; however, all of them were unaware of the study aims. Thus, it is likely that any misclassification would be non-differential and would bias associations towards the null. Liver biopsy is invasive, expensive and unfeasible to perform in a large, generally healthy population. Non-invasive fibrosis markers, such as FIB-4 and NFS, have been validated as acceptable diagnostic tools to identify biopsy-proven advanced liver fibrosis (Angulo *et al*., 2007; Shah *et al*., 2009). A second limitation of this study is that the depressive symptoms were evaluated using a self-administered questionnaire, instead of a physician-determined diagnosis. However, these types of questionnaires are useful in population-based research. In addition, the CES-D is one of the most widely used instruments to measure depression and has been shown to have good reliability and validity for depression assessment in a wide range of populations (Luckett *et al*., 2010). Third, information regarding past medical history and lifestyle behaviours were also collected through self-administered questionnaires, which may have allowed for measurement error. Some degree of residual confounding pertaining to measurement errors and other unmeasured factors cannot be excluded in the associations observed between depressive symptoms and HS. Finally, our study subjects mostly consisted of young and middle-aged, relatively healthy Koreans (of a predominantly single ethnicity), with high accessibility to health care who participated in regular health examinations. Therefore, our findings may not be generalisable to populations of other ethnicities and different demographic features.

## Conclusion

In this large cohort study, depression was positively and independently associated with incident HS and HS plus high probability of advanced fibrosis in individuals with obesity. Further research is needed to determine whether the assessment and treatment of depression helps to prevent HS and related fibrosis, and ultimately helps improve NAFLD prognosis, especially among individuals with obesity.

## Data Availability

The data are not available to be shared publicly because we do not have a permission from the IRB to distribute the data. However, analytical methods are available from corresponding author on a reasonable request.

## Supplementary material

For supplementary material accompanying this paper visit http://dx.doi.org/10.1017/S204579602000116X. click here to view supplementary material
