# Supplementary material for: Depression and increased risk of non-alcoholic fatty liver disease in individuals with obesity
Source: Epidemiol Psychiatr Sci. 2021 Mar 12;30:e23. doi: 10.1017/S204579602000116X (PMC8061301; doi:10.1017/S204579602000116X)
Supplement: Supplementary file 1 [file S204579602000116Xsup001.docx]

**Supplementary materials**

**Supplementary Table 1.** Cumulative incidence rates and risk of incident hepatic steatosis plus high probability of advanced fibrosis based on NAFLD fibrosis score according to CES-D score category in all, non-obese and obese individuals

| CES-D score category | Person-years (PY) | Incident  cases | Incidence  (per 10^3^ PY) | Cumulative Incidence  (per 10^3^ person) | | Age-sex adjusted HR^a^ (95% CI) | Multivariable-adjusted HR^a^ (95% CI) | |
| --- | --- | --- | --- | --- | --- | --- | --- | --- |
|  |  |  |  | 2-Year | 5-Year |  | Model 1 | Model 2 |
| Overall **(n=**142,005**)** |  |  |  |  |  |  |  |  |
| <8 | 415,530.5 | 73 | 0.2 | 0.0 | 0.4 | 1.00 (reference) | 1.00 (reference) | 1.00 (reference) |
| 8-15 | 174,685.6 | 34 | 0.2 | 0.1 | 0.2 | 1.17 (0.78-1.76) | 1.24 (0.82-1.87) | 1.25 (0.83-1.89) |
| ≥16 | 78,782.2 | 17 | 0.2 | 0.0 | 0.1 | 2.06 (1.21-3.51) | 2.04 (1.19-3.49) | 2.06 (1.20-3.53) |
| *P* for trend |  |  |  |  |  | 0.306 | 0.013 | 0.012 |
| Non-obese (n=124,794) |  |  |  |  |  |  |  |  |
| <8 | 363,657.2 | 45 | 0.1 | 0.0 | 0.3 | 1.00 (reference) | 1.00 (reference) | 1.00 (reference) |
| 8-15 | 154,320.9 | 23 | 0.1 | 0.0 | 0.2 | 1.27 (0.77-2.10) | 1.35 (0.81-2.23) | 1.39 (0.84-2.30) |
| ≥16 | 70,854.2 | 8 | 0.1 | 0.0 | 0.0 | 1.49 (0.70-3.18) | 1.44 (0.68-3.08) | 1.45 (0.68-3.11) |
| *P* for trend |  |  |  |  |  | 0.211 | 0.193 | 0.169 |
| Obese (n=17,211) |  |  |  |  |  |  |  |  |
| <8 | 51,873.2 | 28 | 0.5 | 0.2 | 1.4 | 1.00 (reference) | 1.00 (reference) | 1.00 (reference) |
| 8-15 | 20,364.8 | 11 | 0.5 | 0.2 | 0.2 | 1.01 (0.50-2.03) | 1.08 (0.54-2.17) | 1.07 (0.53-2.16) |
| ≥16 | 7,928.0 | 9 | 1.1 | 0.0 | 0.8 | 3.06 (1.44-6.49) | 3.01 (1.41-6.41) | 3.30 (1.55-7.05) |
| *P* for trend |  |  |  |  |  | 0.024 | 0.020 | 0.014 |

Note: *P=0.277* for the overall interaction between obesity and CES-D score category for incident hepatic steatosis plus high NAFLD fibrosis score (model 1).

^a^ Estimated from parametric proportional hazard models. Multivariable model was adjusted for age, sex, center, year of screening exam, education level, body mass index, smoking status, physical activity, alcohol consumption, total energy intake, diabetes, hypertension, and history of cardiovascular disease; model 2: model 1 plus adjustment for systolic blood pressure, glucose, total cholesterol, triglyceride, HDL-C, HOMA-IR and hs-CRP.

Abbreviations: NAFLD, nonalcoholic fatty liver disease; CES-D, Center for Epidemiologic Studies-Depression; CI, confidence interval; HR, hazard ratio; HDL-C, high density lipoprotein cholesterol; HOMA-IR, homeostasis model assessment of insulin resistance; hs-CRP, high sensitivity C-reactive protein.

**Supplementary Table 2.** Risk of incident hepatic steatosis or hepatic steatosis plus high probability of advanced fibrosis based on FIB-4 according to CES-D score category in overall, non-obese and obese individuals using time-dependent analyses (n=142,005)

| CES-D score category | **HR (95% CI)^a^ in model using time-dependent variables** | |
| --- | --- | --- |
|  | **for incident hepatic steatosis** | **for incident hepatic steatosis plus high probability of advanced fibrosis based on FIB-4** |
| Overall |  |  |
| <8 | 1.00 (reference) | 1.00 (reference) |
| 8-15 | 1.07 (1.04-1.10) | 1.23 (0.82-1.83) |
| ≥16 | 1.10 (1.06-1.15) | 1.10 (0.57-2.14) |
| *P* for trend | <0.001 | 0.462 |
| Non-obese |  |  |
| <8 | 1.00 (reference) | 1.00 (reference) |
| 8-15 | 1.05 (1.01-1.08) | 1.25 (0.78-1.99) |
| ≥16 | 1.06 (1.01-1.12) | 1.17 (0.56-2.46) |
| *P* for trend | 0.001 | 0.416 |
| Obese |  |  |
| <8 | 1.00 (reference) | 1.00 (reference) |
| 8-15 | 1.09 (1.04-1.15) | 1.18 (0.54-2.55) |
| ≥16 | 1.20 (1.11-1.30) | 0.86 (0.20-3.67) |
| *P* for trend | <0.001 | 0.925 |
| *P for interaction* | 0.020 | 0.933 |

^a^ Estimated from parametric proportional hazard models with CES-D score category, smoking status, body mass index, alcohol consumption, physical activity, total energy intake, diabetes, hypertension, and history of cardiovascular disease as time-dependent categorical variables and baseline age, sex, center, year of screening exam, and education level as time-fixed variables.

Abbreviations: CES-D, Center for Epidemiologic Studies-Depression; FIB-4, Fibrosis-4 index; CI, confidence interval; HR, hazard ratio.

**Supplementary Table 3.** Risk of incident hepatic steatosis plus high probability of advanced fibrosis based on FIB-4 (≥3.25) according to CES-D score category in all, non-obese and obese individuals

| CES-D score category | Person-years (PY) | Incident  cases | Incidence  (per 10^3^ PY) | Multivariable-adjusted HR^a^ (95% CI) |
| --- | --- | --- | --- | --- |
|  |  |  |  |  |
| Overall **(n=**142,005**)** |  |  |  |  |
| <8 | 415,530.5 | 50 | 0.12 | 1.00 (reference) |
| 8-15 | 174,685.6 | 25 | 0.14 | 1.25 (0.77-2.04) |
| ≥16 | 78,782.2 | 9 | 0.11 | 2.51 (0.73-3.09) |
| *P* for trend |  |  |  | 0.191 |
| Non-obese (n=124,794) |  |  |  |  |
| <8 | 363,657.2 | 40 | 0.11 | 1.00 (reference) |
| 8-15 | 154,320.9 | 17 | 0.11 | 1.06 (0.60-1.88) |
| ≥16 | 70,854.2 | 5 | 0.07 | 0.98 (0.38-2.50) |
| *P* for trend |  |  |  | 0.938 |
| Obese (n=17,211) |  |  |  |  |
| <8 | 51,873.2 | 10 | 0.19 | 1.00 (reference) |
| 8-15 | 20,364.8 | 8 | 0.39 | 2.08 (0.82-5.28) |
| ≥16 | 7,928.0 | 4 | 0.50 | 3.55 (1.11-11.38) |
| *P* for trend |  |  |  | 0.020 |

Note: *P=0.181* for the overall interaction between obesity and CES-D score category for incident hepatic steatosis plus high NAFLD fibrosis score (model 1).

^a^ Estimated from parametric proportional hazard models. Multivariable model was adjusted for age, sex, center, year of screening exam, education level, body mass index, smoking status, physical activity, alcohol consumption, total energy intake, diabetes, hypertension, and history of cardiovascular disease

Abbreviations: NAFLD, nonalcoholic fatty liver disease; FIB-4, Fibrosis-4 index; CES-D, Center for Epidemiologic Studies-Depression; CI, confidence interval; HR, hazard ratio

**Supplementary Table 4.** Risk of incident hepatic steatosis or incident hepatic steatosis plus high probability of advanced fibrosis based on FIB-4 according to CES-D score category in all, non-obese and obese individuals after excluding 75,819 subjects with binge drinking^b^

| CES-D score category | Multivariable-adjusted HR^a^ (95% CI) | |
| --- | --- | --- |
|  | For incident hepatic steatosis | For incident hepatic steatosis plus high probability of advanced fibrosis based on FIB-4 |
| Overall (n=66,186) |  |  |
| <8 | 1.00 (reference) | 1.00 (reference) |
| 8-15 | 1.04 (0.99-1.09) | 1.07 (0.48-2.38) |
| ≥16 | 1.04 (0.98-1.11) | 2.42 (0.95-6.18) |
| *P* for trend | 0.088 | 0.134 |
| Non-obese (n=60,895) |  |  |
| <8 | 1.00 (reference) | 1.00 (reference) |
| 8-15 | 1.02 (0.97-1.07) | 1.04 (0.43-2.55) |
| ≥16 | 1.00 (0.92-1.07) | 1.37 (0.39-4.76) |
| *P* for trend | 0.823 | 0.676 |
| Obese (n=5,291) |  |  |
| <8 | 1.00 (reference) | 1.00 (reference) |
| 8-15 | 1.08 (0.98-1.19) | 1.27 (0.21-7.72) |
| ≥16 | 1.27 (1.12-1.45) | 8.37 (1.64-42.86) |
| *P* for trend | <0.001 | 0.026 |
| *P for interaction* | 0.005 | 0.194 |

^a^ Estimated from parametric proportional hazard models. Multivariable model was adjusted for age, sex, center, year of screening exam, education level, body mass index, smoking status, physical activity, alcohol consumption, total energy intake, diabetes, hypertension, and history of cardiovascular disease

^b^ Binge drinking was defined by any response other than “never” to the question “How often do you have 6 or more drinks per occasion?”

Abbreviations: FIB-4, Fibrosis-4 index; NAFLD, nonalcoholic fatty liver disease; CES-D, Center for Epidemiologic Studies-Depression; CI, confidence interval; HR, hazard ratio

**Supplementary Table 5.** Risk of incident hepatic steatosis or incident hepatic steatosis plus high probability of advanced fibrosis based on FIB-4 according to CES-D score category in all, non-obese and obese individuals after further excluding 3,230 subjects taking medication associated with hepatic steatosis or psychiatric mediation during follow-up

| CES-D score category | Multivariable-adjusted HR^a^ (95% CI) | |
| --- | --- | --- |
|  | For incident hepatic steatosis | For incident hepatic steatosis plus high probability of advanced fibrosis based on FIB-4 |
| Overall (n=66,186) |  |  |
| <8 | 1.00 (reference) | 1.00 (reference) |
| 8-15 | 1.03 (1.004-1.06) | 1.38 (0.94-2.02) |
| ≥16 | 1.05 (1.01-1.10) | 1.70 (0.97-2.98) |
| *P* for trend | 0.002 | 0.134 |
| Non-obese (n=60,895) |  |  |
| <8 | 1.00 (reference) | 1.00 (reference) |
| 8-15 | 1.02 (0.99-1.06) | 1.24 (0.80-1.93) |
| ≥16 | 1.00 (0.95-1.05) | 1.22 (0.60-2.47) |
| *P* for trend | 0.508 | 0.355 |
| Obese (n=5,291) |  |  |
| <8 | 1.00 (reference) | 1.00 (reference) |
| 8-15 | 1.05 (0.99-1.11) | 1.92 (0.91-4.07) |
| ≥16 | 1.24 (1.15-1.34) | 3.41 (1.33-8.74) |
| *P* for trend | <0.001 | 0.007 |
| *P for interaction* | <0.001 | 0.202 |

^a^ Estimated from parametric proportional hazard models. Multivariable model was adjusted for age, sex, center, year of screening exam, education level, body mass index, smoking status, physical activity, total energy intake, diabetes, hypertension, and history of cardiovascular disease

Abbreviations: FIB-4, Fibrosis-4 index; CES-D, Center for Epidemiologic Studies-Depression; CI, confidence interval; HR, hazard ratio
